# Variations in neonatal pain management following web-based continuing education: An explanatory sequential mixed-methods study across 15 neonatal intensive care units

**DOI:** 10.1016/j.ijnsa.2026.100719

**Published:** 2026-10-23

**Authors:** Mio Kojima, Sayo Fukushima

**Affiliations:** Department of Fundamental Nursing, Division of Advanced Nursing Practice, Graduate School of Biomedical & Health Sciences, Hiroshima University, Hiroshima, Japan

**Keywords:** Education, Nursing, Continuing, Evidence-based practice, Diffusion of innovation, Interprofessional relations, Intensive care units, Neonatal, Pain management, Quality improvement

## Abstract

**Background:**

Despite growing evidence supporting neonatal pain management, its implementation in neonatal intensive care units remains inconsistent. Though web-based continuing education can improve nurses’ knowledge of neonatal pain management, the organisational and collaborative processes through which acquired knowledge is translated into routine clinical practice remain poorly understood.

**Objective:**

To identify organizational and interprofessional factors associated with the successful implementation of neonatal pain management following a web-based continuing education programme in Japanese neonatal intensive care units and explain variations in implementation outcomes across facilities.

**Design:**

Explanatory sequential mixed-methods study**.**

**Setting(s):**

Fifteen neonatal intensive care units incorporated a web-based continuing education program on neonatal pain assessment and non-pharmacological pain management into their annual nursing education plans.

**Participants:**

Quantitative data were collected from all units. Qualitative data were obtained through semi-structured interviews with 25 participating nurses.

**Methods:**

In the quantitative phase, longitudinal facility-level data were analysed to evaluate changes in neonatal pain assessment and non-pharmacological pain management practices before and after the programme implementation. The facilities were subsequently classified according to implementation outcomes. In the qualitative phase, semi-structured interviews were conducted with participants to explore their experiences of neonatal pain management and interprofessional collaboration following the programme implementation. Quantitative and qualitative findings were integrated to explain variations in implementation outcomes across facilities.

**Results:**

Implementation outcomes varied substantially across the participating units. Integrated analysis demonstrated that facilities with higher implementation levels at 12 months were characterised by proactive physician-nurse communication before procedures, collaborative pain management during procedures, shared recognition of the importance of neonatal pain management, nurse-led practice, sharing pain assessment and records, and family participation in pain relief interventions. These factors were associated with the translation of educational knowledge into routine clinical practice.

**Conclusions:**

Educational interventions alone were insufficient to improve neonatal pain management. Successful implementation may be associated with collaborative organizational processes through which acquired knowledge is translated into routine practice. Physician-nurse communication before invasive procedures may be associated with the translation of educational knowledge into practice.

**Registration:**

Not registered.

**Social media abstract:**

Same e-learning, different results. Better implementation of neonatal pain management was linked to interprofessional communication.

**What is already known**

• Evidence-based neonatal pain management remains inconsistently implemented.
• Web-based education improves knowledge, but practices vary.
• Mechanisms linking education to implementation are poorly understood.

**What this paper adds**

• Implementation rates varied substantially across 15 neonatal intensive care units.
• Physician–nurse communication was linked to higher pain management implementation.
• Collaborative practice may translate educational knowledge into action.

## 1 Background

Neonates cannot verbally communicate their pain. Furthermore, severe pain among preterm infants with limited physiological reserves may paradoxically result in diminished or absent behavioural responses, including reduced facial expression and motor activity (Breton-Piette et al., 2024). Pain assessment is especially challenging in critically ill infants who receive sedation and mechanical ventilation following surgery because behavioural indicators of pain may be masked, necessitating healthcare professionals’ accurate assessment (Lago et al., 2025; Shimizu et al., 2025).

Historically, these unique characteristics have contributed to the misconception about a high pain threshold and little or no analgesic management for neonates. However, researchers have demonstrated that neonates experience significant pain and that untreated pain may adversely affect both short- and long-term health and neurodevelopmental outcomes (Williams and Lascelles, 2020).

Evidence of the neurobiological vulnerability of neonates emphasises the importance of effective pain management. During early development, tactile input transmitted by Aβ fibres and nociceptive input transmitted by C fibres converge within the dorsal horn of the spinal cord (Puchalski and Hummel, 2002). Under typical developmental conditions, the predominance of nonpainful tactile stimulation contributes to the maturation of sensory pathways and enables the discrimination between touch and pain. However, infants admitted to neonatal intensive care units are repeatedly exposed to painful stimuli that are uncommon during normal development, such as diagnostic procedures, therapeutic interventions, surgery, and disease-related pain. Without systematic assessment and management of pain as a part of routine clinical care, repeated pain exposure may negatively influence developmental trajectories.

Over recent decades, evidence for neonatal pain management has expanded considerably, and validated pain assessment tools and non-pharmacological interventions are now widely recommended (Committee on Fetus and Newborn and Section on Anesthesiology and Pain Medicine, 2016; Committee for Guidelines on Neonatal Pain Prevention and Management, 2025). However, the implementation of evidence-based pain management in neonatal intensive care units remains suboptimal. Recent reviewers reported that more than three decades of research have shown limited improvement in the frequency of painful procedures and the provision of pain-relieving interventions (Bueno et al., 2024). Needle-related procedures remain the most common source of procedural pain and considerable variation persists in the reporting and delivery of pain-relief strategies. To address this evidence-practice gap, responsibility for pain management must extend beyond individual clinicians to encompass organizational systems that support practice change (Bueno et al., 2024).

One strategy for promoting evidence-based practice is continuing professional education. However, in neonatal intensive care units, the implementation of tools for the assessment of neonatal pain remains limited, partly because of insufficient educational resources and training opportunities (Ismail et al., 2024). Web-based learning has become increasingly common in healthcare settings because it offers flexible access to educational content regardless of time and location (Regmi and Jones, 2020). Researchers conducting a systematic review have reported that e-learning improved knowledge, confidence, and attitudes in continuing education (Dentice et al., 2026). However, evaluations of continuing nursing education have been based more on learner satisfaction and knowledge outcomes than on the translation of acquired knowledge into clinical practice and care-quality improvements.

Researchers applying Kirkpatrick's evaluation model found that e-learning improved nurses’ knowledge and skills but provided limited evidence for the transfer of learning into clinical practice and patient care outcomes (Rouleau et al., 2019). Similarly, researchers studying neonatal intensive care unit nurses have reported improvements in the knowledge of neonatal pain assessment and competence in using pain-assessment tools following e-learning interventions (Campbell-Yeo et al., 2022; Ozawa et al., 2022a, 2022b). However, how this newly acquired knowledge is embedded in routine clinical practice remains poorly understood.

Implementation science has increasingly emphasised that educational interventions should be evaluated not only for their effects on knowledge acquisition but also for their ability to facilitate changes in clinical practice (Skivington et al., 2021; Damschroder et al., 2022). Multifaceted implementation strategies combining education, audits and feedback, clinical champions, and interprofessional collaboration may enhance evidence-based nursing practice (Fontaine et al., 2024). Institution-wide initiatives in neonatal pain management have been shown to improve the quality of pain management (Stevens et al., 2025). However, because such interventions comprise multiple interacting components, determining mechanisms that contribute to successful changes in practice is difficult (Lewis et al., 2020).

In particular, little is known about processes through which knowledge acquired through educational interventions is translated into practice or about the organizational and interprofessional factors that facilitate these processes. Previous researchers have suggested that the impact of educational interventions varies across institutions under the influence of organizational culture, communication patterns, and support from managers and colleagues (Rouleau et al., 2019). While education can improve knowledge, there is limited evidence about how knowledge is transformed into routine clinical practice. Understanding this knowledge-to-practice transition is a central concern in the field of implementation science. Since close collaboration between nurses and physicians is required for neonatal pain management, organizational communication and interprofessional working relationships are crucial for determining whether educational gains are translated into improved clinical care.

Therefore, we aimed to explore why differences in neonatal pain management, with a particular focus on pain assessment and non-pharmacological pain relief during such skin-breaking procedures as blood sampling, have emerged across institutions in Japan following the implementation of a web-based continuing education programme. Specifically, we sought to identify the organizational and interprofessional implementation factors associated with the translation of acquired knowledge into routine neonatal pain prevention and management. By integrating quantitative and qualitative findings in this mixed-methods study, we aimed to explain how educational interventions contributed to practice changes in neonatal intensive care unit settings.

## 2 Methods

### 2.1 Study design

A mixed-methods approach based on an explanatory sequential mixed-methods design was employed (Creswell and Plano Clark, 2018) to explain the between-institution differences in neonatal pain management identified through quantitative results. An explanatory sequential mixed-methods design was used to understand the factors associated with the observed outcomes by interpreting and integrating qualitative data into quantitative findings.

In the quantitative phase, longitudinal institution-level data were collected to examine changes in pain assessment and non-pharmacological pain relief practices before and after the implementation of a web-based educational programme. In the qualitative phase, semi-structured interviews were conducted with neonatal intensive care unit nurses to explore their experiences with neonatal pain management and collaboration following the educational intervention.

Subsequently, variability in implementation outcomes across institutions was identified through quantitative analyses, and the institutions were classified into higher-implementation at 12 months and lower-implementation at 12 months groups. To explain the between-institutional differences identified in the quantitative findings, comparative analyses were conducted between the higher-implementation at 12 months and lower-implementation at 12 months groups using qualitative data. Furthermore, when integrating and interpreting the qualitative findings in relation to the differences in implementation outcomes identified in the quantitative results, the principles of the “following a thread” approach (O’Cathain et al., 2010) guided the analysis by using the identified between-institution differences in implementation outcomes as the organising thread for comparing and interpreting the qualitative data. Finally, quantitative and qualitative findings were integrated and interpreted to examine the organizational and interprofessional factors associated with the translation of knowledge acquired through education into neonatal pain management.

### 2.2 Settings and participants

In November 2021, invitations were sent to the directors of nursing, who oversee nursing practice hospital-wide including in the neonatal intensive care unit, at hospitals with regional perinatal medical centres throughout Japan (280 institutions) to participate in the study as collaborating institutions. Consequently, 17 institutions expressed interest, and 15 were ultimately registered as collaborating sites.

Following approval through a centralised ethics review conducted by the lead institution and the receipt of local institutional permission, this multicentre study was conducted in 15 neonatal intensive care units that had incorporated a web-based neonatal pain management educational programme into their annual nursing education plans.

In the quantitative phase, institution-level aggregated data collected from all participating units were included at three time points: before the implementation of the programme (baseline), 6 months after implementation, and 12 months after implementation. These time points were defined in the existing database used for data collection. Data were retrospectively extracted from electronic medical records for a designated 24 h period (00:00–23:59) at each time point and entered into an information technology system (Database © Hiroshima University). In the qualitative phase, neonatal intensive care unit nurses who voluntarily agreed to participate in semi-structured interviews were recruited after they completed the educational programme.

### 2.3 Web-based continuing education intervention

The web-based neonatal pain management educational programme used in this study, e-Pain Management of Neonates (e-PMaN), was previously developed and evaluated through a pilot study and a randomised controlled trial, demonstrating its usability and educational effectiveness among neonatal intensive care unit nurses (Ozawa et al., 2017, 2022a, 2022b). The programme included modules on neonatal pain and pain measurement and assessment and was designed as a self-paced learning programme accessible via internet-connected devices. The modules combined explanatory text, illustrations, movies, short knowledge and skill-check quizzes covering the physiology of neonatal pain and the use of validated pain-assessment tools; participants progressed through the content independently, without required real-time interaction with instructors or peers. Full details of the programme content and delivery are provided in the previous reports (Ozawa et al., 2022a, 2022b). In this study, the participating units incorporated the e-learning programme (e-PMaN) into their annual continuing nursing education programmes for approximately 1 year. Participants individually accessed the programme as part of their routine continuing-education activities.

### 2.4 Quantitative phase

#### 2.4.1 Data collection

Quantitative data were collected from the 15 participating units between March 2022 and May 2023 at three time points: before implementation of the web-based educational programme (baseline), 6 months after implementation, and 12 months after implementation (28 March 2022, 26 September 2022, and 27 March 2023, respectively). Institution-level aggregated data were retrospectively extracted from electronic medical records for a designated 24-hour period (00:00–23:59) at each time point.

Data were collected by nurses or nurse managers designated as collaborators at each participating unit; these collaborators had no financial or personal conflicts of interest related to this study. To ensure consistency across institutions, a standardised data extraction procedure and electronic data entry system developed for quality improvement in neonatal pain care were used. Details of the database system and data collection procedures have been previously reported (Ozawa et al., 2017; Ozawa, 2025). The collected information included the characteristics of hospitals and neonatal intensive care units, organizational infrastructure for neonatal pain management, and pain management related to skin-breaking procedures.

#### 2.4.2 Outcome measures

Skin-breaking procedures were selected as the focus of the outcome measures because they represent the most frequent source of procedural pain in neonatal intensive care units and are the procedures most consistently documented in electronic medical records (Bueno et al., 2024). The primary outcomes were the rates of pain assessment (quality indicator 1: QI 1) and non-pharmacological pain relief (quality indicator 2: QI 2) during skin-breaking procedures. The pain-assessment rate was calculated by dividing the number of skin-breaking procedures for which pain assessment was documented by the total number of skin-breaking procedures. Pain assessment was considered to have been performed if any pain-related assessment or evaluation was documented in the electronic medical record, including the use of standardised pain assessment tools and narrative documentation or institution-specific assessment records.

The rate of non-pharmacological pain relief was calculated by dividing the number of skin-breaking procedures accompanied by at least one non-pharmacological intervention by the total number of skin-breaking procedures. Skin-breaking procedures included heel lancing, peripheral venipuncture, peripheral intravenous catheter insertion, lumbar puncture, arterial catheter insertion, and peripherally inserted central catheter insertion. Data on organizational infrastructure related to neonatal pain management, such as the availability of pain assessment tools, pain management guidelines, pain documentation systems, designated pain education personnel, and organizational audits related to pain management, were also collected.

#### 2.4.3 Data analysis

Descriptive statistics were used to summarise quantitative findings. Continuous variables were reported as means and standard deviations (SD) or medians and interquartile ranges, as appropriate. Categorical variables were summarised using frequencies and percentages. Changes in implementation outcomes and organizational characteristics were descriptively examined across three measurement time points (baseline, 6 months, and 12 months). No missing institution-level data were found during the study period. Given the small number of the participating units (*n* = 15) and the use of institution-level aggregated data, the focus was on describing implementation patterns and identifying differences across units in the quantitative phase.

Additionally, the participating institutions were classified into higher-implementation at 12 months and lower-implementation at 12 months groups based on the level of neonatal pain management practice reached at 12 months after the implementation of the web-based continuing-education programme. This classification reflects the level of implementation reached at 12 months rather than the magnitude of change from baseline. Given the limited number of participating institutions, the classification based on the median was adopted to enable relative grouping according to the implementation outcomes. Using the median values of QI 1 and QI 2 at 12 months after implementation as cut-off values, institutions with both indicators at or above the median were classified as “higher-implementation at 12 months” (*n* = 7). Institutions with one or both indicators below the median were classified as “lower-implementation at 12 months” (*n* = 8). These classifications were performed after the qualitative interviews and the initial transcription process. Thus, qualitative data were collected independent of quantitative data-based classification, and comparisons between groups were introduced only during the qualitative content analysis.

### 2.5 Qualitative phase

#### 2.5.1 Participant recruitment

Semi-structured interviews were conducted with neonatal intensive care unit nurses who had completed the e-PMaN programme. Recruitment was conducted through unit managers, who circulated study information to eligible nurses at each participating institution; nurses who wished to participate then contacted the principal investigator directly. The participants were recruited and interviewed before analysing the quantitative results and classifying the institutional implementation levels. This approach enabled participant recruitment without the influence of quantitative findings and minimised sampling bias. A total of 25 nurses working in 11 of the 15 participating units took part in the study, and all provided written and verbal consent; no eligible nurses volunteered to participate at the remaining four units during the recruitment period. Participants received a book voucher worth JPY 2000 as compensation for their participation. The inclusion criteria were employment at a participating institution and completion of an e-PMaN educational programme. No exclusion criteria were applied.

#### 2.5.2 Data collection

The principal investigator interviewed the participants either online or face-to-face at the participating institutions or universities between May and October 2023. Each participant took part in an individual interview lasting approximately 30–60 min in a quiet environment, either at home or in their workplace. The interview guide (Supplementary Material 1) was used to explore the participants’ previous experiences with pain-management education, educational roles within the neonatal intensive care unit, experiences with the e-PMaN programme, and their perceptions of changes in knowledge and practice following the programme and of the influence of the educational intervention on colleagues, interprofessional collaboration, and neonatal pain management. All interviews were audio-recorded with participants’ consent and transcribed verbatim for analysis. When the interviews were conducted, implementation outcomes had not yet been analysed, and the interviewer was unaware of the future classification of the institutions. Consequently, the interview questions were formulated with a focus on the participants’ experiences related to pain-management education, collaboration, and changes in clinical practice, rather than on the success or failure of implementation. This approach enabled participants to speak freely about their implementation experiences without the influence of quantitative findings and minimised confirmation bias during data collection.

#### 2.5.3 Data analysis

To explore factors that might explain differences in implementation outcomes of neonatal pain management, we focused on differences in pain perceptions among professional groups, as suggested by previous researchers (Yonezawa et al., 2025). Narratives on collaborative situations in pain care between physicians and nurses, nurses and nurses, and nurses and families were selected for analysis. Two researchers (Kojima and Fukushima) independently extracted descriptions related to collaboration in pain care from the interview transcripts and determined excerpts to be included in the analysis through discussion and consensus. Subsequently, a qualitative content analysis following Mayring's summarising content analysis approach was conducted (Mayring, 2022). Relevant text passages were systematically paraphrased, condensed while preserving their original meaning, and inductively organised into subcategories and categories. Categories were generated inductively from data without reference to a preexisting theoretical framework. Interview transcripts were repeatedly reviewed to identify units of meanings related to collaboration among nurses, physicians, and families. As a result, 147 units of meaning related to physician-nurse collaboration, 136 units of meaning related to nurse-nurse collaboration, and 12 units of meaning related to nurse-family collaboration were identified, yielding 295 units of meaning for analysis. Categories were constructed inductively from the entire dataset and subsequently compared across institutions, demonstrating different implementation outcomes in the quantitative data.

The first researcher (Kojima) conducted the initial content analysis, and a second researcher (Fukushima) with expertise in perinatal and neonatal nursing reviewed the analysis. To enhance trustworthiness, the two researchers repeatedly discussed coding decisions and category definitions until they reached a consensus. Inductive content analysis was conducted using all data included in the analysis. Subsequently, representative narratives from the higher-implementation at 12 months and lower-implementation at 12 months institutional groups were extracted. In addition, the co-occurrence network was analysed using KH Coder version 3 (Higuchi, 2016) to complement the content analysis. The second researcher (Fukushima) identified words with equivalent meanings, and discussed with the first researcher (Kojima) regarding whether they could be represented by a single term. Words that appeared at least five times were included in the analysis, and co-occurrence relationships were visualised using the Jaccard coefficient. To examine the structural characteristics of the descriptions related to collaboration, separate network maps consisting of the 60 strongest co-occurrence relationships were generated. These thresholds were selected to minimise visual complexity while facilitating the interpretation of the network structure. The co-occurrence network was analysed to visualise relationships among words appearing in narratives concerning collaboration and compared the structural characteristics of narratives between the higher-implementation at 12 months and lower-implementation at 12 months groups of institutions.

### 2.6 Integration of quantitative and qualitative findings

Quantitative and qualitative findings were integrated using a joint display based on an explanatory sequential mixed-methods design by the authors. Quantitative findings, categories derived from the qualitative content analysis, and results of the co-occurrence network analysis were compared and examined during the integration. Specifically, relationships between the level of neonatal pain management implementation identified in the quantitative phase and the characteristics of collaboration identified through qualitative content analysis were examined. Furthermore, the extent to which the word connection structures were identified through co-occurrence network analysis supported the findings of the qualitative content analysis. Finally, a joint display integrating quantitative findings, qualitative content analysis, and co-occurrence network analysis was developed, and meta-inferences regarding collaborative factors associated with higher level of implementation at 12 months in neonatal pain care were derived. Because the qualitative content analysis and the co-occurrence network analysis were both based on the same interview data, they were treated as complementary rather than independent sources of triangulation. By comparing and interpreting the findings from these two analyses in relation to the quantitative findings, we examined the consistency of the overall interpretation. The meta-inferences were derived by identifying collaborative factors that were consistently associated with the quantitative implementation outcomes and were supported by both qualitative content analysis and co-occurrence network analysis.

### 2.7 Ethical considerations

Ethical approval was obtained from the Epidemiological Research Ethics Committee of Hiroshima University before the commencement of the study (approval no. E2021-2776). Quantitative data were collected from the existing medical records and anonymised before entering the information technology system. Information about the study was provided to parents through notices displayed in the participating units, and parents were given the opportunity to opt out of the study. For the interview, a participant information sheet was provided individually to eligible nurses, and the written and verbal informed consent was obtained from all participants. Participation was voluntary, and the participants were informed that they could withdraw from the study at any time without disadvantage, even after providing their consent.

## 3 Results

### 3.1 Participant characteristics

There were 147 neonatal intensive care unit beds and 367 neonatal intensive care unit nurses across all 15 participating institutions. Ten institutions also had a growing care unit (a unit for neonates who do not require intensive care, admitted directly or transferred from the neonatal intensive care unit after the acute phase), with 132 beds and 170 nurses. The mean ± SD per institution was 9.8 ± 4.4 neonatal intensive care unit beds and 24.5 ± 9.7 neonatal intensive care unit nurses (*n* = 15), and 13.2 ± 5.1 growing care unit beds and 17.0 ± 5.9 growing care unit nurses (*n* = 10) (Supplementary Material 2). The number of hospitalised infants, procedures, and quality-indicator rates at the three measurement time points are presented in Supplementary Material 3.

Organizational changes related to neonatal pain management were also observed during the study period. The proportion of units using a standardised pain-assessment tool increased from 20.0% at baseline to 46.7% at 12 months. The proportion of institutions with established pain-management procedures increased from 6.7% to 13.3%, and the proportion of institutions that could document pain assessments in electronic medical records increased from 26.7% to 60.0%.

Physicians routinely performed blood sampling at all participating institutions. Nurses performed blood sampling only for procedures in which only small blood volumes, such as blood glucose testing, are required. No statistically significant differences in these organizational characteristics were observed between the institutions that were subsequently classified as higher-implementation at 12 months (*n* = 7) or lower-implementation at 12 months (*n* = 8).

A total of 25 nurses from 11 units (six higher-implementation at 12 months institutions and five lower-implementation at 12 months institutions) participated in the interviews. Of these participants, 17 were employed at institutions that were subsequently classified as higher-implementation at 12 months institutions and eight were employed at lower-implementation at 12 months institutions. No marked differences were observed between the two groups in terms of the years of nursing experience or managerial positions, although university graduates were identified only in the group of higher-implementation at 12 months institutions. The participants had a mean of 9.1 ± 7.7 years of neonatal intensive care unit/growing care unit experience and a mean of 14.0 ± 8.7 years of overall nursing experience. Their designations were staff nurses (*n* = 16), charge nurses (*n* = 2), assistant nurse managers (*n* = 5), and nurse managers (*n* = 2). Their educational background included a university degree (*n* = 9), junior college diploma (*n* = 4), and high school nursing qualification (*n* = 12). Four participants were certified neonatal intensive care nurses.

### 3.2 Quantitative findings

Gradual changes in neonatal pain care practices are presented in Fig. 1 and Supplementary Material 3. Overall, the rates of pain assessment and non-pharmacological pain relief showed increasing trends following the implementation of the web-based educational program. However, substantial variations in the magnitude of the changes were observed across institutions. Some institutions demonstrated marked improvements in both indicators over a 12-month period, whereas others showed little change. Several institutions maintained a rate of 0% for both indicators throughout the study period. Fig. 1 illustrates the heterogeneity of the implementation outcomes across institutions. Although all institutions received the same educational intervention, substantial differences in the implementation outcomes were observed. These findings suggest that besides the educational content, organizational factors and collaborative processes supporting implementation may have contributed to improvements in practice. Based on these findings, qualitative analyses focusing on collaboration in neonatal pain care were conducted to gain a deeper understanding of factors associated with differences in implementation outcomes.

**Fig. 1:**
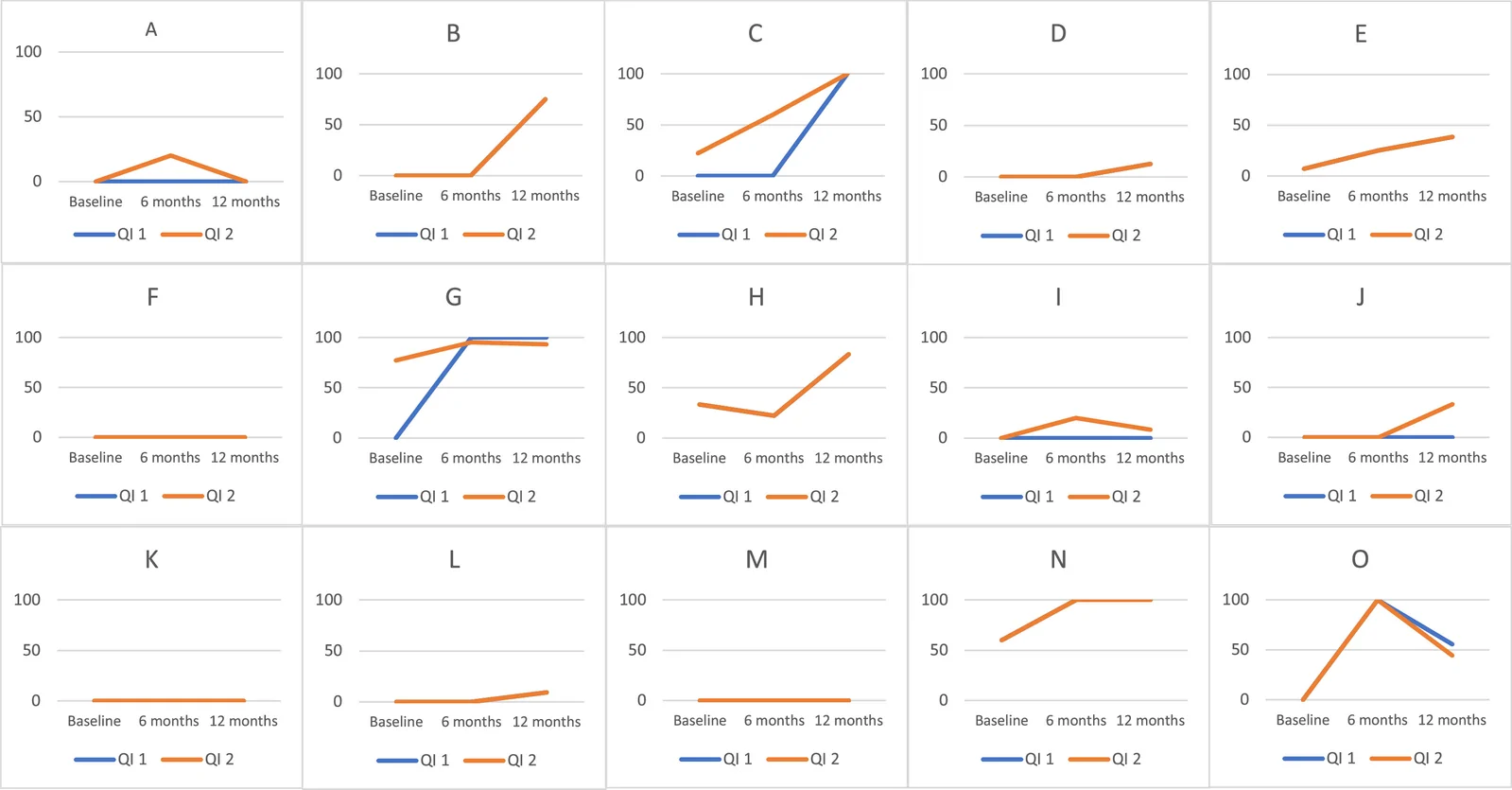
Changes over time in the rates of pain assessment (QI 1) and non-pharmacological pain relief (QI 2) across the 15 participating neonatal intensive care units (panels A-O, one panel per institution). Where the QI 1 line is not visible within a panel, QI 1 and QI 2 values were identical at every time point, and the QI 2 line is directly superimposed on, and therefore obscures, the QI 1 line. Abbreviations: QI 1, quality indicator 1; QI 2, quality indicator 2.

### 3.3 Qualitative findings

In qualitative content analysis, six categories related to collaboration in neonatal pain management were generated (Table 1).

**Table1:** Collaboration between health care professionals and family members in neonatal pain management.

Category	Sub-category
Pre-procedure communication	Pre-procedure communication from physicians to nurses
	Ease of communication between nurses and physicians
Collaborative practice during procedures	Collaboration between nurses and physicians during blood sampling
	Non-pharmacological palliative care provided by nurses
Building a common understanding within the healthcare team	Effects on infants through collaborative practice
	Physicians’ perceptions
	Nurses’ perceptions
Nurse-led promoting practice among nurses	Activities of local champions
	Study sessions and learning activities
Sharing pain assessment and records	Use of pain scales
	Standardization of records
Family-involved pain care	Participation in facilitated tucking
	Procedures during holding and kangaroo care
	Promoting a sense of security for family members
	Parental absence

The participants described collaboration between nurses and physicians as an important factor influencing the implementation of pain management. Communication before the procedures provided opportunities for nurses to prepare and implement non-pharmacological pain relief interventions. In several units, the participants reported that advance notifications of procedures planned by physicians (hereafter referred to as “calling out”) facilitated nurses’ participation in pain management. Participants also described pre-existing approachable relationships between physicians and nurses as factors that facilitated such communication. The participants further described collaborative practices during the procedures, such as holding, use of non-nutritive sucking, and positioning. Some participants reported that family members participated in such pain relief practices as holding their infants during blood-sampling procedures.

A comparison of the higher-implementation at 12 months and lower-implementation at 12 months institutional groups revealed clear differences in collaboration characteristics (Table 2). In the group of higher-implementation at 12 months institutions, active communication between physicians and nurses, shared recognition of the importance of pain care, and the collaborative implementation of pain-relief interventions during procedures were repeatedly described. Initiatives, including those led by certified nurses and education coordinators, were also found as factors promoting the integration of pain management into routine care. Furthermore, families were positioned as active collaborators in pain management. In the group of higher-implementation at 12 months institutions, collaboration involved multiple professional groups and stakeholders including physicians, nurses, and family members.

**Table 2:** Comparison between the higher-implementation at 12 months and lower-implementation at 12 months institutional groups in terms of collaboration categories in neonatal pain management.

Category	Typical comments from the higher-implementation at 12 months group	Typical comments from the lower-implementation at 12 months group	Interpretation of differences between groups
Pre-procedure communication	Doctors would call out to nurses, which served as a trigger for pain-relief interventions. “When the doctor says, ‘I’m going to draw blood,’ the nurse goes to the baby. If the nurse doesn’t come, the doctor will call out to her, saying, “I am drawing blood, so please come.’ So, I think pain-relief measures are always implemented when the nurse assists with blood draws.” (ID 23).	Despite accounts of initiating communication, the connection to pain management practices was limited. “Young doctors probably feel bad about asking because the nurses look so busy, especially in the morning, so they can’t bring themselves to say anything.” (ID 10). “I told the doctors not to just go ahead and insert the needle without saying anything—let’s do this together” (ID 20).	In the group showing higher-implementation, verbal communication served as the starting point for collaborative action, leading to pain relief interventions during procedures.
Collaborative practice during procedures	During blood draws and invasive procedures, nurses implemented pain-relief interventions such as holding the baby and offering a pacifier. “The doctor and a nurse—or two nurses—would perform procedures together. The nurses handled basics, such as facilitated tucking and pacifier sucking” (ID 5).	Collaboration during procedures was limited, and there was little connection to pain relief interventions. “Sometimes I realize they’ve already started the puncture before I even notice, so I’m trying to be more mindful of that myself.” (ID 1)	In the group showing higher-implementation, collaboration was translated into actual pain-care practice.
Building a common understanding within the healthcare team	Colleagues and doctors exchanged views and discussed pain care. “I do think there’s been an increase in discussions like, ‘We have a blood draw coming up, so let’s do it this way’” (ID 8).	Participants mentioned differences in perception and difficulties in obtaining cooperation with colleagues and physicians. “I’ve wanted to do something about pain for a long time and have been studying on my own, but those around me—including doctors, of course—weren’t really interested, and there wasn’t just an atmosphere where people were eager to take action” (ID 20)	In the group showing higher-implementation, pain care had become a shared team goal, whereas in the group of lower-implementation, there was insufficient shared understanding.
Nurse-led promoting practice among nurses	Program coordinators and liaison nurses promoted practices. “Certified nurses and senior nurses specializing in pain care would directly observe blood draws I was in charge of, and afterward, we’d have opportunities to review the procedure and go over the records together.” (ID 14).	There were no accounts regarding the process of applying what they had learned to practice or the activities of those responsible for promoting these initiatives.	The difference lay in the presence or absence not of educational activities but of roles to drive practice and continuous engagement.
Sharing pain assessment and records	Practice was shared using pain-scale assessments and documentation. “We’d ask things like, ‘What level of pain is this?’ and assess it as a group.” (ID 3).	There were no accounts regarding the use of pain scales or pain documentation.	In the group showing higher-implementation, evaluation results were utilised as learning resources for the team.
Family-involved pain care	Family members were positioned as active participants in pain care. “We had the mother hold the baby while we checked blood-glucose levels” (ID 8). “We drew blood while the mother was doing kangaroo care” (ID 9). “Seeing the blood draw go smoothly puts the mother’s mind at ease” (ID 9).	No accounts regarding family involvement were identified.	In the group showing higher-implementation, the scope of collaboration extended beyond healthcare professionals to family members.

In contrast, although participants belonging to the group of lower-implementation at 12 months institutions described educational activities and efforts to raise awareness, their narratives on the collaborative implementation of pain relief interventions were limited. Collaborative practices remained temporary and individual in nature, with the limited involvement of multiple professionals and family members. There was comparatively little evidence for such practices being embedded in routine interprofessional activities, and implementation relied largely on individual efforts.

### 3.4 Co-occurrence network analysis

The results of the co-occurrence network analysis supported the findings obtained through qualitative-content analysis and complemented their structural characteristics (Fig. 2).

**Fig. 2:**
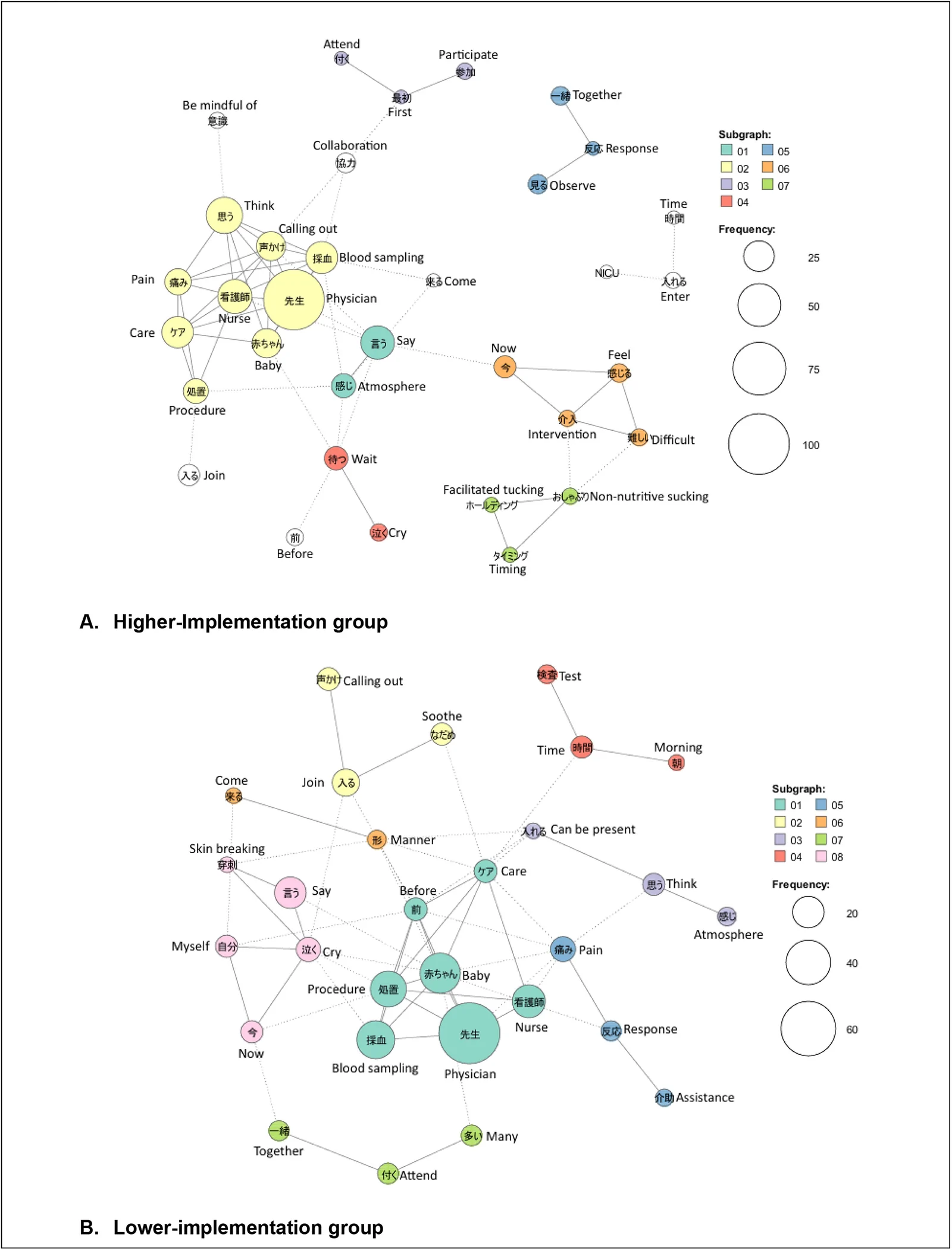
Co-occurrence networks of interview narratives on collaboration in neonatal pain management, by institutional implementation level: (A) higher-implementation group; (B) lower-implementation group. Node size reflects word frequency and node colour indicates sub-graph membership; edges represent co-occurrence relationships among words appearing at least five times in the interview transcripts, visualised using the Jaccard coefficient (KH Coder version 3; Higuchi, 2016).

In the group of higher-implementation at 12 months institutions, words such as “physician”, “blood sampling”, “calling out”, “nurse”, “baby”, and “care” were strongly connected. The co-occurrence relationships among these words suggested that communication before and collaborative practice during the procedures were the central characteristics of pain management in this group. In contrast, in the group of lower-implementation at 12 months institutions, the network was primarily composed of word clusters centred on “nurse”, “baby”, and “care”. Although words related to pain care and procedures appeared in the network, explicit connections indicating collaboration with physicians or collaborative pain-relief practices were limited. The overall network structure suggests that pain management was discussed primarily within the framework of nursing practice rather than as a shared interprofessional practice. Taken together, the co-occurrence network analysis indicated that interprofessional connections were stronger in the group of higher-implementation at 12 months institutions and that collaborative pain management was implemented in a more integrated manner. These findings support the results of the qualitative content analysis.

### 3.5 Integration of quantitative and qualitative findings

Table 3 shows the integrated findings of analyses of quantitative and qualitative content and co-occurrence network. Quantitative analysis revealed substantial variations in neonatal pain management across institutions following the implementation of a web-based continuing-education program. While some institutions reached high rates of pain assessment and non-pharmacological pain-relief by 12 months, others showed little change. During the qualitative content analysis, the higher-implementation at 12 months institutions were characterised by communication before and collaborative practice during procedures; shared recognition of pain care; nurse-led promotion of practice; sharing pain assessment and records; and collaboration with family members. Furthermore, the co-occurrence-network analysis demonstrated strong connections among words related to physicians, nurses, communication, and procedural-pain care, supporting the findings of the content analysis. Integration of these findings suggests that higher implementation levels at 12 months may be associated with the development of collaborative practice structures that connect knowledge acquired through education with routine clinical practice. In particular, communication between physicians and nurses before the procedures was associated with the implementation of specific pain-relief interventions.

**Table 3:** Integrated interpretation of quantitative findings, qualitative categories, and co-occurrence-network analysis related to higher levels of implementation at 12 months in neonatal pain management.

Quantitative findings	Qualitative findings (Mayring)	Co-occurrence network (KH Coder)	Meta-inference
Higher quality implementation rate at 12 months	Communication prior to procedure	Doctor–blood sampling–Verbal communication–nurse	Formation of collaborative practice
	Joint practice among healthcare professions	Blood sampling–care–pain	Implementation of pain management
	Collaboration with families	Timing–facilitated tucking	A culture of pain management that includes family members
Lower quality implementation rate at 12 months	Focus on educational activities	Nurse–procedure–baby–care	Being limited to knowledge verification and sharing and not being practiced

## 4 Discussion

Organizational and collaborative factors associated with the implementation of neonatal pain management following the introduction of a web-based continuing education program within the annual nursing education plans in units were examined in this mixed-methods study. Quantitative findings demonstrated substantial variation among the participating institutions in pain assessment and non-pharmacological pain-relief practices, despite all institutions receiving the same educational intervention. Furthermore, the integration of quantitative and qualitative findings revealed that institutions with higher implementation levels at 12 months were distinguished by interprofessional collaboration around painful procedures, which we discuss in turn below. We suggest that educational interventions alone are insufficient to improve neonatal pain care and that collaborative practice structures may be required to connect knowledge acquired through education with routine clinical practice. These findings are consistent with the Knowledge-to-Action Framework (Graham et al., 2006; Field et al., 2014) and the ‘Normalisation Process Theory’. In particular, according to the Normalisation Process Theory, ‘collective action’ amongst healthcare professionals is essential for successfully integrating new practices into routine care and for implementing and sustaining them in daily clinical practice (May et al., 2009, 2018).

One of the most important findings of this study concerns the role of communication between physicians and nurses prior to invasive procedures. Whilst previous research has shown that collaboration between doctors and nurses is a key determinant of pain management in neonates, the ways in which such collaboration facilitates implementation had not been fully elucidated. We suggest that the collaborative process between nurses and doctors may be a key factor in the ‘transition from nurses’ knowledge to practice’. Specifically, participants from higher-implementation at 12 months institutions repeatedly described advance notifications of planned procedures from physicians to nurses, informal verbal communication, and approachable relationships between these two professional groups. Such communication enabled nurses to prepare and implement non-pharmacological pain-relief interventions, including holding, positioning, and non-nutritive sucking before procedures. By contrast, these forms of communication were reported less frequently in lower-implementation at 12 months institutions.

These findings are particularly important for neonatal care in Japan. In contrast to the adult setting, where blood sampling and vascular access are part of the routine duties of Japanese nurses, in many Japanese neonatal intensive care units, physicians primarily perform invasive procedures, such as blood sampling and vascular access, whereas nurses typically assume supportive roles in pain management. Consequently, nurses’ participation in procedural pain management depends heavily on timely communication from physicians. Furthermore, researchers have highlighted differences between professional groups in their perceptions of infant pain (Yonezawa et al., 2025), and these differences in the division of roles and perceptions of pain are making it difficult to establish a shared approach to pain management challenging.

Furthermore, the Japanese term 先生 (sensei), which appears as a prominent, high-frequency node labelled "Physician" in the co-occurrence networks (Fig. 2), reflects the terminology used by interview participants: Japanese nurses commonly refer to physicians as sensei ("doctor" or "teacher") rather than by their names. This convention reflects the professional authority traditionally associated with physicians and provides a tangible example of the hierarchical power gradient in hospital settings. Therefore, whether physicians and nurses perceive their relationships as approachable and conducive to communication is likely to influence collaborative practice. Although nursing in Japan is regarded as a socially valuable profession, nurses have limited professional authority and decision-making autonomy in medical care compared with physicians; under Article 37 of the Act on Public Health Nurses, Midwives, and Nurses, nurses may not perform medical interventions except under the instruction of a physician or dentist (Act on Public Health Nurses, Midwives, and Nurses, 1948). Additionally, nursing qualifications can be obtained through a wide range of educational pathways from secondary-level nursing programmes to university degrees, resulting in a relatively weak association between educational attainment and professional qualifications. In contrast, in many other countries, neonatal nurses independently undertake a broader range of invasive procedures, potentially reducing their reliance on physician communication to implement pain-relief practices.

Therefore, we suggest that professional role delineation and healthcare system characteristics influence how educational knowledge is translated into practice. Implementation strategies are not universally applicable; instead, these should be designed in accordance with specific organizational and professional contexts. The factors identified in this study, particularly advance notifications before procedures and approachable inter-professional relationships, may represent context-dependent features arising from the division of professional roles within Japanese neonatal intensive care units. For those working in healthcare systems where nurses hold greater procedural autonomy, these specific communication behaviours may be less critical; however, the broader principle, that structural and relational factors between professions shape whether educational gains are translated into practice, is likely to extend beyond the Japanese context. We have also highlighted the importance of promoting nurse-led practices. In higher-implementation at 12 months institutions, nurses, including participants, serving as link nurses, educators, or in other leadership roles played important roles in disseminating knowledge, encouraging colleagues, and maintaining organizational attention to neonatal pain management. These findings are consistent with the implementation science literature and demonstrate the importance of local champions in promoting evidence-based practices (Ozawa, 2025; Bueno et al., 2026). Within the Consolidated Framework for Implementation Research, champions are recognised as key implementation facilitators that promote intervention uptake and sustainability within organisations (Damschroder et al., 2009, 2022).

We have shown the important role of nurses in disseminating knowledge acquired through education and supporting the maintenance of practice within their units. Although web-based educational programmes can enhance individual knowledge, sustained support and local implementation leaders may be necessary to embed this knowledge into routine clinical practice. We have also emphasised the importance of shared recognition of healthcare professionals about neonatal pain management. Higher-implementation at 12 months institutions were characterised by both communications regarding individual procedures and shared beliefs among physicians and nurses regarding the value of pain assessment and pain-relief interventions. Previous studies reported that differences in healthcare professionals’ perceptions of pain can act as barriers to neonatal pain management (Neshat et al., 2023). We suggest that successful implementation is facilitated by the recognition of pain not as the responsibility of particular individuals but as a shared clinical priority for the entire healthcare team.

Family participation in care has emerged as a characteristic of higher-implementation at 12 months institutions. Participants described situations of parents actively participating in such pain-relief practices as holding their infants during blood-sampling procedures. Family participation has been increasingly recognised as an important component of family-centred care and is associated with improved outcomes for both infants and family members (He et al., 2026). Although references to family involvement were less frequent than descriptions of interprofessional collaboration, family participation may contribute to a more comprehensive approach to neonatal pain management.

From a nursing management perspective, neonatal pain management may require more than just educational resources and guidelines incorporated into ward-based annual education plans. Systems that facilitate communication, organizational cultures that support interprofessional collaboration, and environments that enable nurse-led practice promotion may be equally important. Notably, structural resources, such as pain-assessment tools and documentation systems, alone were insufficient to explain differences in implementation outcomes between institutions. Rather, the nature of everyday collaborative practices differed between higher-implementation at 12 months and lower-implementation at 12 months units. Consequently, nurse managers who promote evidence-based neonatal pain management should consider focusing both on the provision of educational programmes and on fostering communication pathways and collaborative organizational cultures.

### 4.1 Limitations

This study has several limitations. First, the classification of institutions into higher-implementation at 12 months and lower-implementation at 12 months groups was based on the level of implementation reached at 12 months rather than on the magnitude of change from baseline; consequently, an institution with high baseline performance but little subsequent change could have been classified as higher-implementation at 12 months, whereas an institution that improved substantially from a low baseline could have been classified as lower-implementation at 12 months. Second, the number of participating institutions was relatively small, limiting the scope of statistical comparisons between institutions, and each institution contributed data from only a single designated 24-hour period at each time point; the resulting proportions may therefore be sensitive to small denominators, particularly at institutions with few skin-breaking procedures. The numerators, denominators, and 95% confidence intervals for each institution and time point are provided in Supplementary Material 4; institution-level comparisons should be interpreted as descriptive. Third, we did not include a control group, and institution-specific programme completion rates were not systematically recorded; therefore, the observed changes and between-institution differences cannot be attributed solely to the web-based education programme. Fourth, the implementation outcomes were evaluated using aggregated institutional-level data rather than patient-level data. Fifth, in the qualitative phase nurses who voluntarily agreed to participate were selected as the participants, and the number of participants from lower-implementation at 12 months institutions was relatively small; the absence of a particular narrative or theme in this group may therefore reflect the limited amount of qualitative data available rather than the genuine absence of that practice. In addition, the participants may have had a greater-than-average interest in neonatal pain management. Sixth, this study was conducted in Japanese neonatal intensive care units, and the findings may reflect the characteristics of the Japanese healthcare system, educational system, and interprofessional division of labour. Therefore, caution should be exercised when generalising or transferring these findings to other countries or healthcare settings.

Despite these limitations, we have provided new insights into collaborative processes associated with higher levels of implementation of neonatal pain care. The integration of quantitative findings, qualitative content analysis, and co-occurrence network analysis suggests that communication between physicians and nurses before procedures was associated with nurses’ translation of knowledge acquired through education into clinical practice. Therefore, future implementation strategies should consider focusing on improving educational content and designing collaborative practice structures to facilitate the application of knowledge in routine clinical care. In particular, organizational processes, such as communication before procedures and champion-led promotion of practice, may be associated with the translation of educational knowledge into practice.

## 5 Conclusions

We observed substantial variation among the participating institutions in the level of implementation of neonatal pain management, following the introduction of a unit-based, web-based continuing nursing education programme. Institutions with higher implementation levels were distinguished by interprofessional collaboration around painful procedures, most notably communication between physicians and nurses before procedures. We suggest that the successful implementation of neonatal pain care may be associated with both educational interventions and organizational and collaborative processes that connect knowledge acquired through education with routine clinical practice.

## Declaration of generative AI and AI-assisted technologies in the manuscript preparation process

During the preparation of this manuscript, the authors used ChatGPT (OpenAI) in order to assist with the translation of the original Japanese manuscript into English and to improve linguistic clarity and readability. After the use of this tool, all content was critically reviewed and revised repeatedly by the authors. The revised manuscript was subsequently proofread by a professional English-language editor and approved by the authors as the final version. The authors take full responsibility for the accuracy, integrity, and completeness of the manuscript.

## Ethics declaration

Written informed consent to take part in the study and to publish the article has been obtained from all participants or their legal representatives. The privacy rights of participants have been observed.

This study was performed in compliance with relevant laws, regulatory frameworks and guidelines where the research took place.

This study was approved by the Epidemiological Research Ethics Committee of Hiroshima University.

(Approval No. E2021-2776)

## Funding

This work was supported by JSPS KAKENHI Grant Numbers JP21K10910, JP17H05108, JP25713066.

## CRediT authorship contribution statement

**Mio Kojima:** Writing – review & editing, Writing – original draft, Visualization, Resources, Project administration, Methodology, Investigation, Funding acquisition, Formal analysis, Data curation, Conceptualization. **Sayo Fukushima:** Writing – review & editing, Visualization, Validation, Project administration, Investigation, Data curation, Formal analysis.

## Declaration of competing interest

The authors declare that they have no known competing financial interests or personal relationships that could have appeared to influence the work reported in this paper.
