# Supplementary material for: Variations in neonatal pain management following web-based continuing education: An explanatory sequential mixed-methods study across 15 neonatal intensive care units
Source: Int J Nurs Stud Adv. 2026 Oct 23;11:100719. doi: 10.1016/j.ijnsa.2026.100719 (PMC13744934; doi:10.1016/j.ijnsa.2026.100719)
Supplement: Supplementary file 1 [file mmc1.docx]

Supplemental 1. Interview Guide and Profiles

1. Self-introduction, name confirmation, estimated interview duration, and procedures

2. Interview procedure

1) Experience of pain duration and content covered in it (individual)

2) Educational role on the ward (organisational)

3) Progress and impact of e-PMaN learning (location, time, what was learned, areas of satisfaction, aspects that were difficult to understand, whether the completion test was conducted, organizational support, motivation, etc.)

4) With the use of e-PMaN as a learning resource, have your own, your colleagues’ or other professionals’ attitudes and behaviours regarding pain care or other aspects of care changed? (Learning, behavioural level, and outcomes)

5) Questions based on changes observed in data entry into the IT system (outcomes).

6) NICU clinical experience and educational background

Note: e-PMaN, e-Pain Management of Neonates.

| Supplemental 2. Background of the participant hospitals at baseline (N=15) | | |  |  |  |  |  |
| --- | --- | --- | --- | --- | --- | --- | --- |
|  |  | n | % | Mean | SD | Median | IQR |
| Organization of hospital management | |  |  |  |  |  |  |
|  | Private | 3 | 20.0 |  |  |  |  |
|  | National/National University | 5 | 33.3 |  |  |  |  |
|  | Private University | 2 | 13.3 |  |  |  |  |
|  | Prefecture/City | 5 | 33.3 |  |  |  |  |
| Number of NICU beds | | 147 |  | 9.8 | 4.4 | 9 |  |
| Number of GCU beds (n=10) | | 132 |  | 13.2 | 5.1 | 12 |  |
| Number of NICU nurses | | 367 |  | 24.5 | 9.7 | 20 |  |
| Number of GCU nurses (n=10) | | 170 |  | 17 | 5.9 | 18.5 |  |
| Number of certified nurses in neonatal care | | 9 |  | 0.6 | 0.6 | 1 |  |
| Number of clinical nurse specialists | | 0 |  | 0 | 0 | 0 |  |
| Number of Full-time physicians | | 95 |  | 6.3 | 4.3 | 5 |  |
| Work schedules for NICU nurses | |  |  |  |  |  |  |
|  | Three-shift system (with equal working hours for all three shifts) | 3 | 20.0 |  |  |  |  |
|  | Two-shift system (with equal working hours for both shifts) | 6 | 40.0 |  |  |  |  |
|  | Irregular three-shift system (with different working hours for the three shifts) | 1 | 6.7 |  |  |  |  |
|  | Irregular two-shift system (with different working hours for the two shifts) | 5 | 33.3 |  |  |  |  |
| Work Schedules for GCU Nurses (n=10) | |  |  |  |  |  |  |
|  | Three-shift system (with equal working hours for all three shifts) | 1 | 10.0 |  |  |  |  |
|  | Two-shift system (with equal working hours for both shifts) | 5 | 50.0 |  |  |  |  |
|  | Irregular three-shift system (with different working hours for the three shifts) | 0 | 0.0 |  |  |  |  |
|  | Irregular two-shift system (a shift system in which the working hours for the two shifts differ) | 4 | 40.0 |  |  |  |  |
| NICU Nursing Models (Multiple answers allowed) | |  |  |  |  |  |  |
|  | Primary nursing | 6 | 40.0 |  |  |  |  |
|  | Team nursing | 4 | 26.7 |  |  |  |  |
|  | Functional nursing model | 1 | 6.7 |  |  |  |  |
|  | Partner Nursing System | 7 | 46.7 |  |  |  |  |
|  | Other | 3 | 20.0 |  |  |  |  |
| NICU Nursing Models* (Multiple answers allowed) | |  |  |  |  |  |  |
|  | Primary nursing | 3 | 30.0 |  |  |  |  |
|  | Team nursing | 4 | 40.0 |  |  |  |  |
|  | Functional nursing model | 1 | 10.0 |  |  |  |  |
|  | Partner Nursing System | 3 | 30.0 |  |  |  |  |
|  | Other | 3 | 30.0 |  |  |  |  |
| Abbreviations: NICU, neonatal intensive care unit; GCU, growing care unit. Notes: GCU is a unit for neonates who do not require intensive care, admitted directly or transferred from the neonatal intensive care unit after the acute phase, broadly comparable to a special care unit in other countries. | | | | | | | |

| Supplemental 3. Descriptive statistics on the number of inpatients and procedures, and implementation rates among 15 NICUs | | | | | | | | | | | | | |  |  |  |  |
| --- | --- | --- | --- | --- | --- | --- | --- | --- | --- | --- | --- | --- | --- | --- | --- | --- | --- |
|  | Baseline | | | | |  | 6 months | | | | |  | 12 months | | | | |
|  | Mean | SD | Median | IQR | Total |  | Mean | SD | Median | IQR | Total |  | Mean | SD | Median | IQR | Total |
| Implementation rate of pain assessments at skin punctures (%) | 6.7 | 16.5 | 0 | 0-0 | ― |  | 23.1 | 39.2 | 0 | 0-25 | ― |  | 38.3 | 41.2 | 12.5 | 0-83.3 | ― |
| Implementation rate of non-pharmacological palliative method at skin punctures (%) | 13.3 | 23.9 | 0 | 0-22.2 | ― |  | 29.5 | 37.9 | 20 | 0-60 | ― |  | 39.8 | 38.6 | 33.3 | 0-83.3 | ― |
| Total number of inpatients | 11.4 | 7.3 | 9 | 5-19 | 172 |  | 11.9 | 8.1 | 12 | 4-19 | 179 |  | 12.2 | 8.5 | 11 | 5-20 | 183 |
| Patients with <28 week-gestation (intubated) | 0.2 | 0.6 | 0 | 0-0 | 3 |  | 0.2 | 0.6 | 0 | 0-0 | 3 |  | 0 | 0 | 0 | 0 | 0 |
| Patients with <28 week-gestation (non-intubated) | 0 | 0 | 0 | 0-0 | 0 |  | 0 | 0 | 0 | 0-0 | 0 |  | 0 | 0 | 0 | 0-0 | 0 |
| Patients with 29–36 week-gestation (intubated) | 0.5 | 0.6 | 0 | 0-1 | 8 |  | 0.1 | 0.4 | 0 | 0-0 | 2 |  | 0.3 | 0.7 | 0 | 0 | 5 |
| Patients with 29–36 week-gestation (non-intubated) | 3.7 | 1.9 | 3 | 2-5 | 56 |  | 3.6 | 3.0 | 4 | 0-6 | 54 |  | 3.1 | 3.2 | 2 | 1-5 | 47 |
| Patients with 37 week-gestation (intubated) | 0.5 | 1.3 | 0 | 0-1 | 8 |  | 0.7 | 1.6 | 0 | 0-1 | 11 |  | 1 | 1.5 | 0 | 0-2 | 15 |
| Patients with 37 week-gestation (non-intubated) | 6.5 | 5.9 | 5 | 2-9 | 97 |  | 0.7 | 1.6 | 0 | 0-1 | 109 |  | 1.5 | 1.5 | 0 | 0-2 | 116 |
| Total number of procedures involving skin puncture | 10.9 | 6.9 | 9 | 4-18 | 164 |  | 7.4 | 5.5 | 6 | 4-10 | 111 |  | 11.2 | 7.6 | 10 | 5-15 | 168 |
| Heel lances | 7.4 | 6.3 | 5 | 3-16 | 111 |  | 5.1 | 5.2 | 3 | 2-5 | 77 |  | 7.1 | 5.3 | 5 | 3-9 | 107 |
| Venous punctures | 3 | 5.1 | 1 | 0-5 | 45 |  | 1.7 | 2.0 | 1 | 0-2 | 26 |  | 2.9 | 3.6 | 2 | 0-4 | 44 |
| Arterial punctures | 0.1 | 0.4 | 0 | 0-2 | 2 |  | 0.1 | 0.4 | 0 | 0-0 | 2 |  | 0.1 | 0.5 | 0 | 0-0 | 2 |
| Lumbar punctures | 0.1 | 0.3 | 0 | 0-0 | 1 |  | 0 | 0 | 0 | 0-0 | 0 |  | 0 | 0 | 0 | 0-0 | 0 |
| Subcutaneous injections | 0.3 | 0.7 | 0 | 0-0 | 5 |  | 0.4 | 1.1 | 0 | 0-0 | 6 |  | 0.9 | 2.4 | 0 | 0-9 | 14 |
| Intramuscular injections | 0 | 0 | 0 | 0-0 | 0 |  | 0 | 0 | 0 | 0-0 | 0 |  | 0.1 | 0.3 | 0 | 0-0 | 1 |
| Thoracic/abdominal punctures | 0 | 0 | 0 | 0-0 | 0 |  | 0 | 0 | 0 | 0-0 | 0 |  | 0 | 0 | 0 | 0-0 | 0 |
| Availability of pain documentation formats on medical records, n(%) | 4(26.7) | ― | ― | ― | ― |  | 5(33.3) | ― | ― | ― | ― |  | 9(60.0) | ― | ― | ― | ― |
| Use of pain measurement tools, n(%) | 3(20.0) | ― | ― | ― | ― |  | 6(40.0) | ― | ― | ― | ― |  | 7(46.7) | ― | ― | ― | ― |
| Pain management protocols available, n(%) | 1(6.7) | ― | ― | ― | ― |  | 2(13.3) | ― | ― | ― | ― |  | 2(13.3) | ― | ― | ― | ― |
| Blood sampling performed by nurses, n(%) | 5(33.3) | ― | ― | ― | ― |  | 5(33.3) | ― | ― | ― | ― |  | 5(33.3) | ― | ― | ― | ― |

**Supplemental 4.** Institution-level numerators (n), denominators (N), and 95% confidence intervals for QI 1 and QI 2 at each time point (15 institutions)

| **Institution** | **Group at 12 months** | **Time point** | **N** | **QI 1: n/N (%) [95% CI]** | **QI 2: n/N (%) [95% CI]** |
| --- | --- | --- | --- | --- | --- |
| A | Lower | Baseline | 20 | 0/20 (0.0%) [0.0–16.1] | 0/20 (0.0%) [0.0–16.1] |
|  |  | 6 months | 10 | 0/10 (0.0%) [0.0–27.8] | 2/10 (20.0%) [5.7–51.0] |
|  |  | 12 months | 16 | 0/16 (0.0%) [0.0–19.4] | 0/16 (0.0%) [0.0–19.4] |
| B | Higher | Baseline | 1 | 0/1 (0.0%) [0.0–79.3] | 0/1 (0.0%) [0.0–79.3] |
|  |  | 6 months | 16 | 0/16 (0.0%) [0.0–19.4] | 0/16 (0.0%) [0.0–19.4] |
|  |  | 12 months | 4 | 3/4 (75.0%) [30.1–95.4] | 3/4 (75.0%) [30.1–95.4] |
| C | Higher | Baseline | 18 | 0/18 (0.0%) [0.0–17.6] | 4/18 (22.2%) [9.0–45.2] |
|  |  | 6 months | 5 | 0/5 (0.0%) [0.0–43.4] | 3/5 (60.0%) [23.1–88.2] |
|  |  | 12 months | 4 | 4/4 (100.0%) [51.0–100.0] | 4/4 (100.0%) [51.0–100.0] |
| D | Lower | Baseline | 17 | 0/17 (0.0%) [0.0–18.4] | 0/17 (0.0%) [0.0–18.4] |
|  |  | 6 months | 4 | 0/4 (0.0%) [0.0–49.0] | 0/4 (0.0%) [0.0–49.0] |
|  |  | 12 months | 8 | 1/8 (12.5%) [2.2–47.1] | 1/8 (12.5%) [2.2–47.1] |
| E | Higher | Baseline | 14 | 1/14 (7.1%) [1.3–31.5] | 1/14 (7.1%) [1.3–31.5] |
|  |  | 6 months | 4 | 1/4 (25.0%) [4.6–69.9] | 1/4 (25.0%) [4.6–69.9] |
|  |  | 12 months | 13 | 5/13 (38.5%) [17.7–64.5] | 5/13 (38.5%) [17.7–64.5] |
| F | Lower | Baseline | 4 | 0/4 (0.0%) [0.0–49.0] | 0/4 (0.0%) [0.0–49.0] |
|  |  | 6 months | 5 | 0/5 (0.0%) [0.0–43.4] | 0/5 (0.0%) [0.0–43.4] |
|  |  | 12 months | 13 | 0/13 (0.0%) [0.0–22.8] | 0/13 (0.0%) [0.0–22.8] |
| G | Higher | Baseline | 22 | 0/22 (0.0%) [0.0–14.9] | 17/22 (77.3%) [56.6–89.9] |
|  |  | 6 months | 21 | 21/21 (100.0%) [84.5–100.0] | 20/21 (95.2%) [77.3–99.2] |
|  |  | 12 months | 15 | 15/15 (100.0%) [79.6–100.0] | 14/15 (93.3%) [70.2–98.8] |
| H | Higher | Baseline | 18 | 6/18 (33.3%) [16.3–56.3] | 6/18 (33.3%) [16.3–56.3] |
|  |  | 6 months | 9 | 2/9 (22.2%) [6.3–54.7] | 2/9 (22.2%) [6.3–54.7] |
|  |  | 12 months | 6 | 5/6 (83.3%) [43.6–97.0] | 5/6 (83.3%) [43.6–97.0] |
| I | Lower | Baseline | 7 | 0/7 (0.0%) [0.0–35.4] | 0/7 (0.0%) [0.0–35.4] |
|  |  | 6 months | 10 | 0/10 (0.0%) [0.0–27.8] | 2/10 (20.0%) [5.7–51.0] |
|  |  | 12 months | 12 | 0/12 (0.0%) [0.0–24.3] | 1/12 (8.3%) [1.5–35.4] |
| J | Lower | Baseline | 4 | 0/4 (0.0%) [0.0–49.0] | 0/4 (0.0%) [0.0–49.0] |
|  |  | 6 months | 2 | 0/2 (0.0%) [0.0–65.8] | 0/2 (0.0%) [0.0–65.8] |
|  |  | 12 months | 3 | 0/3 (0.0%) [0.0–56.2] | 1/3 (33.3%) [6.1–79.2] |
| K | Lower | Baseline | 8 | 0/8 (0.0%) [0.0–32.4] | 0/8 (0.0%) [0.0–32.4] |
|  |  | 6 months | 10 | 0/10 (0.0%) [0.0–27.8] | 0/10 (0.0%) [0.0–27.8] |
|  |  | 12 months | 10 | 0/10 (0.0%) [0.0–27.8] | 0/10 (0.0%) [0.0–27.8] |
| L | Lower | Baseline | 14 | 0/14 (0.0%) [0.0–21.5] | 0/14 (0.0%) [0.0–21.5] |
|  |  | 6 months | 6 | 0/6 (0.0%) [0.0–39.0] | 0/6 (0.0%) [0.0–39.0] |
|  |  | 12 months | 33 | 3/33 (9.1%) [3.1–23.6] | 3/33 (9.1%) [3.1–23.6] |
| M | Lower | Baseline | 9 | 0/9 (0.0%) [0.0–29.9] | 0/9 (0.0%) [0.0–29.9] |
|  |  | 6 months | 6 | 0/6 (0.0%) [0.0–39.0] | 0/6 (0.0%) [0.0–39.0] |
|  |  | 12 months | 17 | 0/17 (0.0%) [0.0–18.4] | 0/17 (0.0%) [0.0–18.4] |
| N | Higher | Baseline | 5 | 3/5 (60.0%) [23.1–88.2] | 3/5 (60.0%) [23.1–88.2] |
|  |  | 6 months | 2 | 2/2 (100.0%) [34.2–100.0] | 2/2 (100.0%) [34.2–100.0] |
|  |  | 12 months | 5 | 5/5 (100.0%) [56.6–100.0] | 5/5 (100.0%) [56.6–100.0] |
| O | Higher | Baseline | 3 | 0/3 (0.0%) [0.0–56.2] | 0/3 (0.0%) [0.0–56.2] |
|  |  | 6 months | 1 | 1/1 (100.0%) [20.7–100.0] | 1/1 (100.0%) [20.7–100.0] |
|  |  | 12 months | 9 | 5/9 (55.6%) [26.7–81.1] | 4/9 (44.4%) [18.9–73.3] |

Abbreviations: CI, confidence interval; N, number of skin-breaking procedures in the 24-hour sampling period; n, number of those procedures for which the indicator was met; QI 1, quality indicator 1 (rate of documented pain assessment); QI 2, quality indicator 2 (rate of non-pharmacological pain relief). Notes: 95% CIs were calculated using the Wilson score method and are descriptive. Institution letters (A–O) correspond to the panels of Figure 1. ‘Higher’ = higher-implementation at 12 months (both QI 1 and QI 2 at or above the median at 12 months [12.5% and 33.3%, respectively]); ‘Lower’ = lower-implementation at 12 months (one or both indicators below the median).
